# Supplementary material for: Coproduction of accessible digital mental health supports in partnership with young people from marginalised backgrounds: a scoping review protocol
Source: BMJ Open. 2024 May 15;14(5):e082247. doi: 10.1136/bmjopen-2023-082247 (PMC11097861; doi:10.1136/bmjopen-2023-082247)
Supplement: Supplementary data [file bmjopen-2023-082247supp002.pdf]

Supplemental file 2

Search of academic database

Pubmed Search

ALL FIELDS Search: ((((("well-being" OR wellbeing OR stress OR "mental disorder" OR "mental illness" OR "mental health" OR depress* OR "psychological health" OR anxiety OR psychiatric OR "mood disorder" OR "mental disease") AND (youth* OR young* OR child* OR adolescen* OR student* OR teen*)) AND (marginali* OR disadvan* OR vulnerab* OR depriv* OR "ethnic minorit*" OR immigra* OR homeless OR minorit* OR "low-income" OR disabili* OR isolat* OR LGBTQ+ OR NEET OR NEETs)) AND (intervention OR promo* OR prevent* OR program* OR support OR polic* OR implementation OR evaluation OR therap* OR develop*)) AND (digital* OR mHealth OR eHealth OR "web-based" OR "internet-based" OR "mobile phone" OR "text message" OR "text-based" OR SMS OR app OR "artificial intelligence" OR tele* OR computeri* OR online OR "electronic health" OR "telemedicine")) AND ("co-produ*" OR "co-design*" OR "youth-led" OR participatory OR collab*) Filters: from 2021 – 2023; **309 returns**

ALL FIELDS Search: ("well-being" OR wellbeing OR stress OR "mental disorder" OR "mental illness" OR "mental health" OR depress* OR "psychological health" OR anxiety OR psychiatric OR "mood disorder" OR "mental disease") AND (youth* OR young* OR child* OR adolescen* OR student* OR teen*) AND (marginali* OR disadvan* OR vulnerab* OR depriv* OR "ethnic minorit*" OR immigra* OR homeless OR minorit* OR "low-income" OR disabili* OR isolat* OR LGBTQ+ OR NEET OR NEETs OR refugee OR indigenous OR neurodiver*) AND (intervention OR promo* OR prevent* OR program* OR support OR polic* OR implementation OR evaluation OR therap* OR develop*) AND (digital* OR mHealth OR eHealth OR "web-based" OR "internet-based" OR "mobile phone" OR "text message" OR "text-based" OR SMS OR app OR "artificial intelligence" OR tele* OR computeri* OR online OR "electronic health" OR "telemedicine") AND ("co-produ*" OR "co-design*" OR "youth-led" OR participatory OR collab*) Filters: from 2021/1/1 - 2023/10/23; 350 returns
