# Supplementary material for: Coproduction of accessible digital mental health supports in partnership with young people from marginalised backgrounds: a scoping review protocol
Source: BMJ Open. 2024 May 15;14(5):e082247. doi: 10.1136/bmjopen-2023-082247 (PMC11097861; doi:10.1136/bmjopen-2023-082247)
Supplement: Supplementary data [file bmjopen-2023-082247supp003.pdf]

Supplemental file 3

Template for charting included articles

**Reference**	
**Country of study**	
**Focus of paper: Co Production in design, implementation, evaluation or overall evaluation of co-designing digital mental health intervention**	
**Population; target issue; brief account of digital intervention; mental health promotion-primary prevention or targeted intervention**	
**Methodology of co-design; sample; outcomes**	
**Key Theme(s)**	
**Issues/ debates identified**	

Limitations	
**Evaluation of study (key findings / overall conclusions only)**	
**Relevance to research objective/s**	
**Notes:**	
**Reviewed by:**	

* This template will be further refined at the review stage and updated accordingly.
