# Supplementary material for: Coproduction of accessible digital mental health supports in partnership with young people from marginalised backgrounds: a scoping review protocol
Source: BMJ Open. 2024 May 15;14(5):e082247. doi: 10.1136/bmjopen-2023-082247 (PMC11097861; doi:10.1136/bmjopen-2023-082247)
Supplement: Author's manuscript [file bmjopen-2023-082247.draft_revisions.pdf]

BMJ Open

BMJ Open is committed to open peer review. As part of this commitment we make the peer review history of every article we publish publicly available.

When an article is published we post the peer reviewers' comments and the authors' responses online. We also post the versions of the paper that were used during peer review. These are the versions that the peer review comments apply to.

The versions of the paper that follow are the versions that were submitted during the peer review process. They are not the versions of record or the final published versions. They should not be cited or distributed as the published version of this manuscript.

BMJ Open is an open access journal and the full, final, typeset and author-corrected version of record of the manuscript is available on our site with no access controls, subscription charges or pay-per-view fees (http://bmjopen.bmj.com).

If you have any questions on BMJ Open's open peer review process please email info.bmjopen@bmj.com

BMJ Open

Co-production of accessible digital mental health supports in partnership with young people from marginalised backgrounds: a scoping review protocol

Journal:	*BMJ Open*
Manuscript ID	bmjopen-2023-082247
Article Type:	Protocol
Date Submitted by the Author:	17-Nov-2023
Complete List of Authors:	Kealy, Carmen; University of Galway<br>Potts, Courtney; Ulster University<br>Donohoe, Gary; University of Galway<br>Barry, Margaret M.; University of Galway<br>Mulvenna, Maurice; University of Ulster, School of Computing<br>O'Neill, Siobhan; Ulster University, Psychology
Keywords:	Adolescent, MENTAL HEALTH, eHealth, Internet

SCHOLARONE™
Manuscripts

Co-production of accessible digital mental health supports in partnership with young people from marginalised backgrounds: a scoping review protocol

Word count: 2,182

Keywords

Adolescent, Mental Health, Mental Health Services, Digital Technology, Health Promotion

Abstract

Introduction Despite evidence supporting the enhancement of youth mental health services through digital tools, there's a lack of guidance on engaging young people in co-production processes during the design and evaluation of these technologies. User input is crucial in digital mental health, especially for disadvantaged, vulnerable, and marginalised youth as they are often excluded from co-production. A scoping review will explore the literature on co-production processes with marginalised youth in the context of digital mental health supports, from mental health promotion to targeted interventions. The review aims to map and summarise evidence, inform the overall research project, and address knowledge gaps.

Methods and analysis The scoping review is guided by Arksey and O'Malley's framework and the Preferred Reporting Items for Systematic Reviews and Meta-analysis Protocols Extension for Scoping Reviews (PRISMA-ScR). Electronic databases will be systematically searched from 2021 onwards including: PubMed, Scopus, EBSCO, ASSIA, Web of Science, Ovid Medline, Cochrane database, Embase, Google Scholar, ProQuest, OAIster and BASE. A range of study designs and evidence that illustrate engagement with marginalised young people (aged 16-25) in design, implementation, and evaluation of digital technologies for young people's mental health, will be considered for inclusion. At least two reviewers will screen full texts and chart data. The results of this scoping review will be summarised quantitatively through numerical counts of included literature and qualitatively through a narrative synthesis.

Ethics and dissemination Ethics approval is not required. Results will be disseminated through publications in peer reviewed journals.

Registration This scoping review protocol has been registered with the Open Science Framework (https://osf.io/9xhgv).

ARTICLE SUMMARY

Strengths and limitations of this study

- This is the first scoping review that explores the most appropriate co-production processes for engaging 'disadvantaged, vulnerable and/or marginalised young people' in the different stages of designing and assessing digital mental health technologies.

For peer review only - http://bmjopen.bmj.com/site/about/guidelines.xhtml

- This study protocol is guided by the methodology framework of Arksey and O'Malley (1) but also the updated version of this framework by Levac and colleagues (2) and will follow a systematic approach to data synthesis (PRISMA-ScR).
- The review will be limited to studies published from 2021 onwards to capture the most current literature on the use of co-production methods and the dynamic nature of this topic.
- Due to the scoping review design, quality assessments of the included articles will not be performed.
- While there may be a shortage of published work in this topic area that could limit the amount of data available, this synthesis of knowledge will serve as a basis for the wider research project and concrete calls to action.

For peer review only

INTRODUCTION

There is increasing evidence that digital mental health supports can improve the accessibility of mental health services and supports for young people, including marginalised youth with mental health needs who would otherwise unlikely seek help (3). Guidelines with regards to digital mental health interventions highlight the importance of early user input in the development, implementation, and evaluation of technologies to ensure that they are engaging, feasible, acceptable, and potentially effective (4). However, there is a lack of guidance concerning the most appropriate co-production processes for engaging young people in the different stages of assessing digital mental health technologies, especially with regard to those who are disadvantaged, vulnerable and /or marginalised (5–7).

Focusing on innovative youth-led digital mental health approaches, this review will consider co-production in the design, development, implementation, and evaluation phases of digital mental health supports for marginalised young people, as well as overall evaluations of co-production processes in this context. The review is part of a larger project, called 'Atlantic Futures' (8). One of the work packages (Research Stream 4), within this project explores accessible digital supports and blended services to promote youth mental health on a shared island basis in Ireland and Northern Ireland. This is to be achieved through engaging with young people (aged 16-25), including those who are marginalised, and the staff who support them in health, youth, and community services.

Marginalisation

This scoping review focuses on young people categorised as 'marginalised' in the digital mental health support context. A briefing paper produced by UNICEF's Office of Research defines "disadvantaged, vulnerable and/or marginalised (DVM) adolescents as individuals aged 10–19, who are excluded from social, economic and/or educational opportunities enjoyed by other adolescents in their community due to numerous factors beyond their control" (9). These factors include those operating at the social level (such as economic inequality, violence, stigma, racism, migration), family level (including neglect and abuse) and individual level (e.g., disability, ethnicity). Examples for DVM youth include immigrants, refugees, sexual minorities, orphans, and those who belong to stigmatised indigenous, ethnic, or religious groups.

Previous reviews on co-production in digital mental health interventions have highlighted an underrepresentation of young people with learning disabilities and specific difficulties (5) and a marked absence of youth samples including, but not limited to migrants, asylum seekers and refugees, those experiencing homelessness and those from socioeconomically deprived backgrounds (10).

In the context of digital mental health technologies, the concept of digital marginalisation is also highly relevant, and this typically refers to people who do not have or use the internet, who lack access to fast and reliable internet connection (e.g., people living in rural areas), and who lack the skills or the access to developing skills for using the internet (10). Many marginalised groups face barriers in accessing online services, which can further perpetuate the "digital divide," a technology-based form of social inequality that can increase marginalisation in other areas of their lives (11).

For peer review only

Considering the above, the project seeks to take into account the specific needs of young people who are disadvantaged, vulnerable and /or marginalised, from a socio-economic, cultural, and digital perspective.

Thus, in order to investigate marginalisation, the scoping review will include, but not be limited to, the following groups of young people.

- Living in isolated rural areas (12)
- Socioeconomically deprived
- Unemployed
- Not in education, employment, or training (NEET)
- Immigrants
- Asylum seekers
- Refugees
- Travellers
- LGBTQ+
- In care/after care
- Disability
- Homeless

This scoping review intends to explore the most appropriate co-production processes for engaging young people in the different stages of designing, developing, implementing and evaluating digital mental health technologies, especially with regard to those who are marginalised. Due to the exploratory nature of this research question, a scoping review approach has been selected to explore the breadth and depth of the extant literature on co-production processes with marginalised youth in the context of digital mental health supports, ranging from mental health promotion to targeted interventions. The review aims to map and summarise the evidence, inform the overall research project, as well as identify and address knowledge gaps (13).

METHODS AND ANALYSIS

This scoping review is prepared in accordance with the Preferred Reporting Items for Systematic Reviews and Meta-analysis Protocols Extension for Scoping Reviews guidelines (PRISMA-ScR) (14). A scoping review is a systematic way of exploring a topic area, in which main concepts and knowledge gaps are identified within a developing field of research[12]. In contrast to systematic reviews, the research questions for a scoping review are frequently broader and more exploratory (14), and hence appropriate for this topic due to insufficient research and a lack of synthesised knowledge. Guided by Arksey and O'Malley's (1) five-step set, but also Levac and colleagues (2) update of this framework, the review process will follow this tentative timeline:

1. Identifying the research question—completed in October 2023

2. Identifying relevant studies—completed in October 2023

3. Study selection—November-December 2023

4. Charting the data—January 2024.

For peer review only - http://bmjopen.bmj.com/site/about/guidelines.xhtml

For peer review only

5. Collating, summarising, and reporting the results—February 2024

Identifying the research question

In keeping with the exploratory nature of scoping reviews, the research team identified one main research question: What are the most appropriate co-production processes for engaging young people in the different stages of designing and assessing digital mental health technologies, especially regarding those who are marginalised?

Identifying relevant studies

Following a preliminary search to see the volume returned, the following electronic databases will be systematically searched: PubMed, Scopus, EBSCO (CINAHL/PsycINFO/PsycArticles), ASSIA (Applied Social Sciences Index & Abstracts), Web of Science, Ovid Medline, Cochrane database, Embase, Google Scholar, ProQuest Dissertations & Theses A&I, OAIster (Catalog of Open Access Resources) and BASE (Bielefeld Academic Search Engine). The focus of this scoping review is informed by previous reviews on co-production and/or marginalised youth in digital mental health interventions (3–7), which have been conducted and/or published since the onset of the global Coronavirus and associated restrictions in March 2020. Highlighting an underrepresentation of marginalised youth, our searches will lead on from these reviews and be limited from January 2021 to the date of search commencement in October 2023, to ensure that the literature reflects the most current literature on the use of co-production methods and thus the nature of this dynamic research topic. Published work from 2021 onwards is included to mirror the current state of literature more accurately, given the accelerated use and assessment of digital mental health technologies throughout the sector during the pandemic (15).

Title, abstract and keyword fields will be searched with the following search terms where advanced search options are possible: (“well-being” OR wellbeing OR stress OR “mental disorder” OR “mental illness” OR “mental health” OR depress* OR “psychological health” OR anxiety OR psychiatric OR “mood disorder” OR “mental disease”) AND (youth* OR young* OR child* OR adolescen* OR student* OR teen*) AND (marginali* OR disadvan* OR vulnerab* OR depriv* OR “ethnic minorit*” OR immigra* OR homeless OR minorit* OR “low-income” OR disabili* OR isolat* OR LGBTQ+ OR NEET OR NEETs) AND (intervention OR promo* OR prevent* OR program* OR support OR polic* OR implementation OR evaluation OR therap* OR develop*) AND (digital* OR mHealth OR eHealth OR “web-based” OR “internet-based” OR “mobile phone” OR “text message” OR “text-based” OR SMS OR app OR “artificial intelligence” OR tele* OR computeri* OR online OR “electronic health” OR “telemedicine”) AND (“co-produ*” OR “co-design*” OR “youth-led” OR participatory OR collab*).

For Google Scholar and BASE (Bielefeld Academic Search Engine) the following search terms will be used: (Co-production OR Co-design) AND (Digital mental health) AND (marginal*youth* OR disadvant* adolescen* OR vulnerable teen* OR support* OR intervention OR promot* OR prevent* OR develop* OR evalua* OR implem*).

An example of the electronic search strategy for PubMed has been included (online supplemental file 1)

For peer review only - http://bmjopen.bmj.com/site/about/guidelines.xhtml

Results from academic and grey literature databases will be downloaded in RIS format and uploaded to the Covidence software (16), a screening and data extraction tool for conducting reviews, which allows screening to be more efficient and easily tracked.

Study selection

Two senior research team members will be assigned the role of reviewers and will screen all articles identified from the databases independently and 20% of the overall article returns will also be screened by two other members of the research team. Titles and abstracts will be reviewed based on the initial search and will be included in accordance with the eligibility criteria described below. Duplicate articles will be removed, while full texts will be examined to create a final list of included studies. Any disagreements on the inclusion of any articles will be resolved through discussion and in consultation with other team members if necessary.

The reference lists of the included papers will be examined as well to verify that all relevant publications are incorporated. As the review develops, other techniques for searching may be implemented, and any additions or changes will be noted.

Eligibility Criteria

Empirical research and discussions in the literature will be included in the review if they: (1) are published since January 2021; (2) are published in English; (3) focus on co-production/co-design of digital youth mental health supports with marginalised young people aged 16-25; and (4) utilise innovative youth-led methodologies to design, develop, implement and/or evaluate digital mental health supports for young people.

Articles will NOT be included if they are: (1) non-digital interventions; (2) technologies developed primarily for physical health or diagnostic, screening, monitoring, communication or data management tools; (3) focus on digital mental health supports without youth input in the development, implementation and/ or evaluation of these supports; and (4) involving children (under the age of 16) and adults (over the age of 25).

Charting the data

Data from the identified studies will be collected and charted according to key groupings, e.g., based on the focus of the article – design, development, implementation, evaluation. From each eligible article, the reviewers will include authorship, year and journal of publication, the general characteristics of participants (i.e., age, gender, and sociodemographic information), geographic location of study, study methods, the identified co-production processes for digital mental health supports, limitations, and any other key findings. An example of a charting template has been included (online supplemental file 2). A combination of Excel and Covidence management software will be used to collate the charted data and to manage the screening process.

Collating, summarising and reporting the results

As this scoping review is intended to provide an overview of the existing literature, and not to critically appraise the included articles, risk of bias will not be assessed. However, we will include a discussion of any limitations found in the included body of evidence. The results of

For peer review only - http://bmjopen.bmj.com/site/about/guidelines.xhtml

this scoping review will be summarised quantitatively by highlighting the amount and type of studies reviewed, and qualitatively through a narrative synthesis. Tables and/or charts will be used to map the study findings and provide an overview, and the screening process will be visualised through a flowchart. Given the exploratory nature of this study, any factors related to co-producing digital mental health supports will be considered for review. Of particular interest is the sourcing of state-of-the-art examples that engage with marginalised young people (aged 16-25) in an innovative way, not only in the initial exploration/design but also implementation and evaluation phase of digital technologies for young people's mental health. The review also seeks to identify published works that have evaluated overall co-production processes in the creation of digital mental health supports for young people.

Patient and public involvement

The design of this scoping review protocol did not involve patients or the public.

Ethics and dissemination

As this scoping review is intended to synthesise the current breadth of knowledge on co-production processes with marginalised youth in the context of digital mental health supports, no ethics approval is required. The results of this scoping review will be disseminated through publications in peer-reviewed journals.

DISCUSSION

The results of this study will establish what is currently known about the most appropriate co-production processes for engaging young people in the different stages of assessing digital technologies, especially with regard to those who are disadvantaged, vulnerable and /or marginalised. To the best of our knowledge, this scoping review protocol is the first on this topic and will identify key themes and gaps in understanding how to engage with marginalised young people (aged 16-25) in an innovative and meaningful way when designing/ developing, as well as implementing and evaluating, digital mental health technologies for young people's mental health. The results will serve as the basis for the wider project, including: a qualitative exploration of digital mental health challenges facing young people across the island of Ireland; identifying, with young people, appropriate digital mental health apps and interventions; validating, in partnership with young people, what digital mental health interventions work; and using these findings to inform policy and practice recommendations for health providers across the island of Ireland and beyond. The review will contribute to existing literature in the field by not only highlighting what has been achieved to date but also by proposing potential ways of advancing the inclusion of marginalised youth in the co-production of digital mental health supports, This will ensure user input, access, and subsequent improvement in digital mental health technologies.

AUTHOR STATEMENT

Author contributors ANONYMOUS FOR REVIEW contributed to the development of this manuscript. ANONYMOUS conceptualised the research question, designed the study and prepared the first draft of the manuscript. ANONYMOUS helped refine the research question and provide review expertise. All authors contributed to the refining of the study design, as

For peer review only - http://bmjopen.bmj.com/site/about/guidelines.xhtml

well as to the editing and revising of this protocol. All authors have approved the final manuscript for submission.

Funding Atlantic Futures is a €4 million four year cross-border research project funded by The North-South Research Programme. The NSRP is a collaborative scheme funded through the Government's Shared Island Fund. It is administered by the Higher Education Authority (HEA) on behalf of the Department of Further and Higher Education, Research, Innovation and Science.

Competing interests None declared.

Patient consent for publication Not required.

For peer review only

For peer review only - http://bmjopen.bmj.com/site/about/guidelines.xhtml

REFERENCES

1. Arksey H, O'Malley L. Scoping studies: Towards a methodological framework. Int J Soc Res Methodol Theory Pract. 2005;8(1):19–32.

2. Levac D, Colquhoun H, O'Brien KK. Scoping studies: advancing the methodology. Implement Sci. 2010;5:69.

3. Piers R, Williams JM, Sharpe H. Review: Can digital mental health interventions bridge the 'digital divide' for socioeconomically and digitally marginalised youth? A systematic review. Child Adolesc Ment Health. 2023;28(1):90–104.

4. Liverpool S, Mota CP, Sales CMD, Čuš A, Carletto S, Hancheva C, et al. Engaging children and young people in digital mental health interventions: Systematic review of modes of delivery, facilitators, and barriers. J Med Internet Res. 2020;22(6).

5. Bevan Jones R, Stallard P, Agha SS, Rice S, Werner-Seidler A, Stasiak K, et al. Practitioner review: Co-design of digital mental health technologies with children and young people. J Child Psychol Psychiatry Allied Discip. 2020;61(8):928–40.

6. Bear HA, Nunes LA, DeJesus J, Liverpool S, Moltrecht B, Neelakantan L, et al. Determination of Markers of Successful Implementation of Mental Health Apps for Young People: Systematic Review. J Med Internet Res. 2022;24(11):1–17.

7. Maxwell N, Corliss C. Implementing co-productive practice with public services: Messages from the research [Internet]. 2020. Available from: https://www.violencepreventionwales.co.uk/cms-assets/research/Implementing-co-productive-practice-with-public-services.pdf

8. Atlantic Futures Project [Internet]. Available from: https://www.atlanticfutures.com/

9. Auerswald CL, Piatt AA, Mirzazadeh A. Research with Disadvantaged, Vulnerable and/or Marginalized Adolescents [Internet]. Innocenti Research Briefs. Innocenti, Florence; 2017. Available from: https://www.violencepreventionwales.co.uk/cms-assets/research/Implementing-co-productive-practice-with-public-services.pdf

10. Eickers G, Rath M. Digital Change and Marginalized Communities: Changing Attitudes Towards Digital Media in the Margins. ICERI2021 Proc. 2021;1:5286–93.

11. Sin J, Franz RL, Munteanu C, Neves BB. Digital design marginalization: New perspectives on designing inclusive interfaces. In: Conference on Human Factors in Computing Systems - Proceedings. Yokohama, Japan: ACM PUB27 New York, NY, USA; 2021.

12. Pobal Maps [Internet]. Available from: https://maps.pobal.ie/WebApps/DeprivationIndices/index.html

13. Peters MDJ, Marnie C, Tricco AC, Pollock D, Munn Z, Alexander L, et al. Updated methodological guidance for the conduct of scoping reviews. JBI Evid Synth. 2020;18(10):2119–26.

14. Tricco AC, Lillie E, Zarin W, O'Brien KK, Colquhoun H, Levac D, et al. PRISMA Extension for Scoping Reviews (PRISMA-ScR): Checklist and Explanation. Ann Intern Med. 2018 Oct;169(7):467–73.

For peer review only - http://bmjopen.bmj.com/site/about/guidelines.xhtml

15. Li J. Digital technologies for mental health improvements in the COVID-19 pandemic: a scoping review. BMC Public Health. 2023;23(1):1–10.

16. Veritas Health Innovation. Covidence systematic review software [Internet]. Melbourne, Australia; Available from: www.covidence.org

For peer review only

For peer review only - http://bmjopen.bmj.com/site/about/guidelines.xhtml

BMJ OPEN- draft protocol Supplementary file 1

Search of academic database

Pubmed searched on 23-10-23

ALL FIELDS Search: ((((("well-being" OR wellbeing OR stress OR "mental disorder" OR "mental illness" OR "mental health" OR depress* OR "psychological health" OR anxiety OR psychiatric OR "mood disorder" OR "mental disease") AND (youth* OR young* OR child* OR adolescen* OR student* OR teen*)) AND (marginali* OR disadvan* OR vulnerab* OR depriv* OR "ethnic minorit*" OR immigra* OR homeless OR minorit* OR "low-income" OR disabili* OR isolat* OR LGBTQ+ OR NEET OR NEETs)) AND (intervention OR promo* OR prevent* OR program* OR support OR polic* OR implementation OR evaluation OR therap* OR develop*)) AND (digital* OR mHealth OR eHealth OR "web-based" OR "internet-based" OR "mobile phone" OR "text message" OR "text-based" OR SMS OR app OR "artificial intelligence" OR tele* OR computeri* OR online OR "electronic health" OR "telemedicine")) AND ("co-produ*" OR "co-design*" OR "youth-led" OR participatory OR collab*) Filters: from 2021 – 2023; **309 returns**

For peer review only

BMJ OPEN- draft protocol Supplementary file 2

Template for charting included articles

**Reference**	
**Country of study**	
**Focus of paper: Co Production in design, implementation, evaluation or overall evaluation of co-designing digital mental health intervention**	
**Population; target issue; brief account of digital intervention; mental health promotion-primary prevention or targeted intervention**	
**Methodology of co-design; sample; outcomes**	
**Key Theme(s)**	
**Issues/ debates identified**	

For peer review only

**Evaluation of study (key findings / overall conclusions only)**	
**Relevance to research objective/s**	
**Notes:**	
**Reviewed by:**	

* This template will be further refined at the review stage and updated accordingly.

For peer review only

BMJ Open

Co-production of accessible digital mental health supports in partnership with young people from marginalised backgrounds: a scoping review protocol

Journal:	*BMJ Open*
Manuscript ID	bmjopen-2023-082247.R1
Article Type:	Protocol
Date Submitted by the Author:	06-Mar-2024

Complete List of Authors:	Kealy, Carmen; University of Galway<br>Potts, Courtney; Ulster University<br>Mulvenna, Maurice; University of Ulster, School of Computing<br>Donohoe, Gary; University of Galway<br>O'Neill, Siobhan; Ulster University, Psychology<br>Barry, Margaret M.; University of Galway

**Primary Subject Heading**:	Mental health
Secondary Subject Heading:	Qualitative research, Health services research
Keywords:	Adolescent, MENTAL HEALTH, eHealth, Internet

SCHOLARONE™
Manuscripts

Co-production of accessible digital mental health supports in partnership with young people from marginalised backgrounds: a scoping review protocol

Carmen Kealy[1], Courtney Potts[2]*, Maurice D. Mulvenna[3], Gary Donohoe[4], Siobhán O'Neill[2] and Margaret M. Barry[1],

[1] Health Promotion Research Centre, University of Galway, Ireland

[2] School of Psychology, Ulster University, Northern Ireland

[3] School of Computing, Ulster University, Northern Ireland

[4] School of Psychology, University of Galway, Ireland

*corresponding author: c.potts@ulster.ac.uk, 2-24 York Street, Belfast, BT15 1AP, tel: +442895367509

Word count: 2,172

Keywords

Adolescent, Mental Health, eHealth, Internet

Abstract

Introduction Despite evidence supporting the enhancement of youth mental health services through digital tools, there's a lack of guidance on engaging young people in co-production processes during the design and evaluation of these technologies. User input is crucial in digital mental health, especially for disadvantaged, vulnerable, and marginalised young people as they are often excluded from co-production. A scoping review will explore the literature on co-production processes with marginalised young people in the context of digital mental health supports, from mental health promotion to targeted interventions. The review is guided by one main research question: what are the most appropriate co-production processes for engaging young people in the different stages of designing and assessing digital mental health technologies, especially regarding those who are marginalised? The review aims to map and summarise evidence, inform the overall research project, and address knowledge gaps.

Methods and analysis The scoping review is guided by Arksey and O'Malley's framework and the Preferred Reporting Items for Systematic Reviews and Meta-analysis Protocols Extension for Scoping Reviews (PRISMA-ScR). From October 22nd to October 24th 2024, electronic databases were systematically searched: PubMed, Scopus, EBSCO, ASSIA, Web of Science, Ovid Medline, Cochrane database, Embase, Google Scholar, ProQuest, OAIster and BASE. Papers from 2021 onwards with a range of study designs and evidence that illustrate engagement with marginalised young people (aged 16-25) in design, implementation, and evaluation of digital technologies for young people's mental health, will be considered for inclusion. At least two reviewers will screen full texts and chart data. The

For peer review only - http://bmjopen.bmj.com/site/about/guidelines.xhtml

results of this scoping review will be summarised quantitatively through numerical counts of included literature and qualitatively through a narrative synthesis.

Ethics and dissemination Ethics approval is not required. Results will be disseminated through publications in peer reviewed journals.

Registration This scoping review protocol has been registered with the Open Science Framework (https://osf.io/9xhgv).

ARTICLE SUMMARY

Strengths and limitations of this study

- This review will explore the literature on co-production across all stages of designing and evaluating digital mental health supports for young people, with a particular emphasis on marginalised young people who are often excluded from these processes.
- The review will be limited to studies published from 2021 onwards to capture the most current literature on the use of co-production methods and the dynamic nature of this topic.
- Due to the scoping review design, quality assessments of the included articles will not be performed.
- While there may be a shortage of published work in this topic area that could limit the amount of data available, this synthesis of knowledge will serve as a basis for the wider research project and concrete calls to action.

For peer review only - http://bmjopen.bmj.com/site/about/guidelines.xhtml

INTRODUCTION

There is increasing evidence that some digital mental health supports may improve the accessibility of mental health services and supports for young people, including marginalised young people with mental health needs who would otherwise unlikely seek help (1). Guidelines with regard to digital mental health interventions highlight the importance of early user input in the development, implementation, and evaluation of technologies to ensure that they are engaging, feasible, acceptable, and potentially effective (2). However, there is a lack of guidance concerning the most appropriate co-production processes for engaging young people in the different stages of assessing digital mental health technologies, especially with regard to those who are disadvantaged, vulnerable and /or marginalised (3-5).

Focusing on innovative digital mental health approaches led by young people, this review will consider co-production in the design, development, implementation, and evaluation phases of digital mental health supports for marginalised young people, as well as overall evaluations of co-production processes in this context. The review is part of a larger project, called ‘Atlantic Futures’ (6). One of the work packages (Research Stream 4), within this project explores accessible digital supports and blended services to promote youth mental health on a shared island basis in Ireland and Northern Ireland. This is to be achieved through engaging with young people (aged 16-25), including those who are marginalised, and the staff who support them in health, youth, and community services.

Marginalisation

This scoping review focuses on young people categorised as ‘marginalised’ in the digital mental health support context. A briefing paper produced by UNICEF’s Office of Research defines “disadvantaged, vulnerable and/or marginalised adolescents as individuals aged 10–19, who are excluded from social, economic and/or educational opportunities enjoyed by other adolescents in their community due to numerous factors beyond their control” (7). These factors include those operating at the social level (such as economic inequality, violence, stigma, racism, migration), family level (including neglect and abuse) and individual level (e.g., disability, ethnicity). Examples for disadvantaged, vulnerable and/or marginalised young people include immigrants, refugees, orphans, and those who belong to stigmatised indigenous, ethnic, or religious groups. They also include individuals who identify as belonging to sexual minorities (e.g., gay, lesbian, bisexual, queer) or gender minorities (e.g., transgender, gender diverse).

Previous reviews on co-production in digital mental health interventions have highlighted either an underrepresentation of young people with learning disabilities and specific difficulties (3) or a marked absence of young people including, but not limited to those who are migrants, asylum seekers and refugees, those experiencing homelessness but also those from socioeconomically deprived backgrounds (8).

In the context of digital mental health technologies, the concept of digital marginalisation is also highly relevant, and this typically refers to people who do not have or use the internet, who lack access to fast and reliable internet connection (e.g., people living in rural areas), and who lack the skills or the access to developing skills for using the internet (8). Many marginalised groups face barriers in accessing online services, which can further perpetuate

For peer review only - http://bmjopen.bmj.com/site/about/guidelines.xhtml

the “digital divide,” a technology-based form of social inequality that can increase marginalisation in other areas of their lives (9).

Considering the above, the project seeks to take into account the specific needs of young people who are disadvantaged, vulnerable and /or marginalised, from a socio-economic, cultural, and digital perspective.

Thus, in order to investigate co-production and marginalisation, the scoping review will include, but not be limited to, the following groups of young people,.who have been identified as marginalised in the Irish context

- Living in isolated rural areas (10)
- Socioeconomically deprived
- Unemployed
- Not in education, employment, or training (NEET)
- Immigrants
- Asylum seekers
- Refugees
- Travellers
- LGBTQ+
- In care/after care
- Disability
- Homeless

This scoping review intends to explore the most appropriate co-production processes for engaging young people in the different stages of designing, developing, implementing, and evaluating digital mental health technologies, especially with regard to those who are marginalised. Due to the exploratory nature of this research question, a scoping review approach has been selected to explore the breadth and depth of the extant literature on co-production processes with marginalised young people in the context of digital mental health supports, ranging from mental health promotion, and primary prevention to targeted interventions. This includes all types of digital interventions, such as websites and apps but not studies that report on digital delivery of services that are typically administered face-to-face such as remote counselling. The review aims to map and summarise the evidence, inform the overall research project, as well as identify and address knowledge gaps (11).

METHODS AND ANALYSIS

This scoping review is prepared in accordance with the Preferred Reporting Items for Systematic Reviews and Meta-analysis Protocols Extension for Scoping Reviews guidelines (PRISMA-ScR) (12) (Supplemental file 1). A scoping review is a systematic way of exploring a topic area, in which main concepts and knowledge gaps are identified within a developing field of research (13). In contrast to systematic reviews, the research questions for a scoping review are frequently broader and more exploratory (13), and hence appropriate for this topic due to insufficient research and a lack of synthesised knowledge. Guided by Arksey and O’Malley’s (13) five-step set, but also Levac and colleagues (14) update of this framework, the review process will follow this tentative timeline:

1. Identifying the research question—completed in October 2023

For peer review only - http://bmjopen.bmj.com/site/about/guidelines.xhtml

2. Identifying relevant studies—completed in October 2023

3. Study selection—November-December 2023

4. Charting the data—January 2024.

5. Collating, summarising, and reporting the results—February 2024

Identifying the research question

In keeping with the exploratory nature of scoping reviews, the research team identified one main research question: what are the most appropriate co-production processes for engaging young people in the different stages of designing and assessing digital mental health technologies, especially regarding those who are marginalised?

Identifying relevant studies

Following a preliminary search to see the volume returned, the following electronic databases will be systematically searched: PubMed, Scopus, EBSCO (CINAHL/PsycINFO/PsycArticles), ASSIA (Applied Social Sciences Index & Abstracts), Web of Science, Ovid Medline, Cochrane database, Embase, Google Scholar, ProQuest Dissertations & Theses A&I, OAIster (Catalog of Open Access Resources) and BASE (Bielefeld Academic Search Engine). The focus of this scoping review is informed by previous reviews on co-production and/or marginalised young people in digital mental health interventions (1-5), which have been conducted and/or published since the onset of the global Coronavirus pandemic and associated restrictions in March 2020. Highlighting an underrepresentation of marginalised young people , searches will lead on from these reviews and be limited from January 2021 to the date of search commencement in October 2023, to ensure that the review reflects the most current literature on the use of co-production methods and thus the nature of this dynamic research topic. Published work from 2021 onwards is included to mirror the current state of literature more accurately, given the accelerated use and assessment of digital mental health technologies throughout the sector during the pandemic (15).

Title, abstract and keyword fields will be searched with the following search terms (Table 1) where advanced search options are possible.

Table 1: Search terms

Mental health	"well-being" OR wellbeing OR stress OR "mental disorder" OR "mental illness" OR "mental health" OR depress* OR "psychological health" OR anxiety OR psychiatric OR "mood disorder" OR "mental disease"
Young people	youth* OR young* OR child* OR adolescen* OR student* OR teen*

For peer review only - http://bmjopen.bmj.com/site/about/guidelines.xhtml

Marginalisation*	marginali* OR disadvan* OR vulnerab* OR depriv* OR “ethnic minorit*” OR immigra* OR homeless OR minorit* OR “low-income” OR disabili* OR isolat* OR LGBTQ+ OR NEET OR NEETs OR refugee OR indigenous OR neurodiver*
Interventions	Intervention OR promo* OR prevent* OR program* OR support OR polic* OR implementation OR evaluation OR therap* OR develop*
Technology	digital* OR mHealth OR eHealth OR “web-based” OR “internet-based” OR “mobile phone” OR “text message” OR “text-based” OR SMS OR app OR “artificial intelligence” OR tele* OR computeri* OR online OR “electronic health” OR “telemedicine”
Co-production	“co-produ*” OR “co-design*” OR “youth-led” OR participatory OR collab*

*Search terms for the category marginalisation consist of definition terms provided by UNICEF’s briefing paper (9) as well as search terms found adequate to capture the groups of young people identified as marginalized.in the Irish but also international context

For peer review only

For Google Scholar and BASE (Bielefeld Academic Search Engine) the following search terms will be used: (Co-production OR Co-design) AND (Digital mental health) AND (marginal*youth* OR disadvant* adolescen* OR vulnerable teen* OR support* OR intervention OR promot* OR prevent* OR develop* OR evalua* OR implem*).

An example of the electronic search strategy for PubMed has been included (supplemental file 2)

Results from academic and grey literature databases will be downloaded in RIS format and uploaded to the Covidence software (16), a screening and data extraction tool for conducting reviews, which allows screening to be more efficient and easily tracked.

Study selection

Two senior research team members will be assigned the role of reviewers and will screen all articles identified from the databases independently and 20% of the overall article returns will also be screened by two other members of the research team. Titles and abstracts will be reviewed based on the initial search and will be included in accordance with the eligibility criteria described below. Duplicate articles will be removed, while full texts will be examined to create a final list of included studies. Any disagreements on the inclusion of any articles will be resolved through discussion and in consultation with other team members if necessary.

For peer review only - http://bmjopen.bmj.com/site/about/guidelines.xhtml

The reference lists of the included papers will be examined as well to verify that all relevant publications are incorporated. As the review develops, other techniques for searching may be implemented, and any additions or changes will be noted.

Eligibility Criteria

Empirical research and discussions in the literature will be included in the review if they: (1) are published since January 2021; (2) are published in English; (3) focus on co-production/co-design of digital youth mental health supports with marginalised young people aged 16-25; and (4) utilise innovative youth-led methodologies to design, develop, implement and/or evaluate digital mental health supports for young people.

Articles will NOT be included if they are: (1) non-digital interventions; (2) technologies developed primarily for physical health or diagnostic, screening, monitoring, communication or data management tools; (3) focus on digital mental health supports without young people's input in the development, implementation and/ or evaluation of these supports; and (4) involving children (under the age of 16) and adults (over the age of 25).

Charting the data

Data from the identified studies will be collected and charted according to key groupings, e.g., based on the focus of the article – design, development, implementation, evaluation. From each eligible article, the reviewers will include authorship, year and journal of publication, the general characteristics of participants (i.e., age, gender, and sociodemographic information), geographic location of study, study methods, the identified co-production processes for digital mental health supports, limitations, and any other key findings relating to the research question. An example of a charting template has been included (supplemental file 3). A combination of Excel and Covidence management software will be used to collate the charted data and to manage the screening process.

Collating, summarising and reporting the results

As this scoping review is intended to provide an overview of the existing literature, and not to critically appraise the included articles, risk of bias will not be assessed. However, we will include a discussion of any limitations found in the included body of evidence. The results of this scoping review will be summarised quantitatively by highlighting the amount and type of studies reviewed, and qualitatively through a narrative synthesis. Tables and/or charts will be used to map the study findings and provide an overview, and the screening process will be visualised through a flowchart. Given the exploratory nature of this study, any factors related to co-producing digital mental health supports will be considered for review. Of particular interest is the sourcing of state-of-the-art examples that engage with marginalised young people (aged 16-25) in an innovative way, not only in the initial exploration/design but also implementation and evaluation phase of digital technologies for young people's mental health. The review also seeks to identify published works that have evaluated overall co-production processes in the creation of digital mental health supports for young people.

Patient and public involvement

The design of this scoping review protocol did not involve patients or the public.

For peer review only - http://bmjopen.bmj.com/site/about/guidelines.xhtml

Ethics and dissemination

As this scoping review is intended to synthesise the current breadth of knowledge on co-production processes with marginalised youth in the context of digital mental health supports, no ethics approval is required. The results of this scoping review will be disseminated through publications in peer-reviewed journals.

DISCUSSION

The results of this study will establish what is currently known about the most appropriate co-production processes for engaging young people in the different stages of assessing digital technologies, especially with regard to those who are disadvantaged, vulnerable and /or marginalised. To the best of our knowledge, this scoping review protocol is the first on this topic and will identify key themes and gaps in understanding how to engage with marginalised young people (aged 16-25) in an innovative and meaningful way when designing/ developing, as well as implementing and evaluating, digital mental health technologies for young people's mental health. The results will serve as the basis for the wider project, including: a qualitative exploration of digital mental health challenges facing young people across the island of Ireland; identifying, with young people, appropriate digital mental health apps and interventions; validating, in partnership with young people, what digital mental health interventions work; and using these findings to inform policy and practice recommendations for health providers across the island of Ireland and beyond. The review will contribute to existing literature in the field by not only highlighting what has been achieved to date but also by proposing potential ways of advancing the inclusion of marginalised young people in the co-production of digital mental health supports, This will ensure user input, access, and subsequent improvement in digital mental health technologies.

AUTHOR STATEMENT

Author contributors CK, CP, MDM, GD, SON and MMB contributed to the development of this manuscript. CK conceptualised the research question, designed the study and prepared the first draft of the manuscript. MMB helped refine the research question and provide review expertise. All authors contributed to the refining of the study design, as well as to the editing and revising of this protocol. All authors have approved the final manuscript for submission.

Funding Atlantic Futures is a €4 million four year cross-border research project funded by The North-South Research Programme (422560). The North-South Research Programme is a collaborative scheme funded through the Government's Shared Island Fund. It is administered by the Higher Education Authority (HEA) on behalf of the Department of Further and Higher Education, Research, Innovation and Science.

Competing interests None declared.

Patient consent for publication Not required.

For peer review only - http://bmjopen.bmj.com/site/about/guidelines.xhtml

REFERENCES

1. Piers R, Williams JM, Sharpe H. Review: Can digital mental health interventions bridge the 'digital divide' for socioeconomically and digitally marginalised youth? A systematic review. Child Adolesc Ment Health. 2023;28(1):90–104.

2. Liverpool S, Mota CP, Sales CMD, Čuš A, Carletto S, Hancheva C, et al. Engaging children and young people in digital mental health interventions: Systematic review of modes of delivery, facilitators, and barriers. J Med Internet Res. 2020;22(6).

3. Bevan Jones R, Stallard P, Agha SS, Rice S, Werner-Seidler A, Stasiak K, et al. Practitioner review: Co-design of digital mental health technologies with children and young people. J Child Psychol Psychiatry Allied Discip. 2020;61(8):928–40.

4. Bear HA, Nunes LA, DeJesus J, Liverpool S, Moltrecht B, Neelakantan L, et al. Determination of Markers of Successful Implementation of Mental Health Apps for Young People: Systematic Review. J Med Internet Res. 2022;24(11):1–17.

5. Maxwell N, Corliss C. Implementing co-productive practice with public services: Messages from the research [Internet]. 2020. Available from: https://www.violencepreventionwales.co.uk/cms-assets/research/Implementing-co-productive-practice-with-public-services.pdf

6. Atlantic Futures Project [Internet]. Available from: https://www.atlanticfutures.com/

7. Auerswald CL, Piatt AA, Mirzazadeh A. Research with Disadvantaged, Vulnerable and/or Marginalized Adolescents [Internet]. Innocenti Research Briefs. Innocenti, Florence; 2017. Available from: https://www.unicef-irc.org/publications/878-research-with-disadvantaged-vulnerable-and-or-marginalized-adolescents.html

8. Eickers G, Rath M. Digital Change and Marginalized Communities: Changing Attitudes Towards Digital Media in the Margins. ICERI2021 Proc. 2021;1:5286–93.

9. Sin J, Franz RL, Munteanu C, Neves BB. Digital design marginalization: New perspectives on designing inclusive interfaces. In: Conference on Human Factors in Computing Systems - Proceedings. Yokohama, Japan: ACM PUB27 New York, NY, USA; 2021.

10. Pobal Maps [Internet]. Available from: https://maps.pobal.ie/WebApps/DeprivationIndices/index.html

11. Peters MDJ, Marnie C, Tricco AC, Pollock D, Munn Z, Alexander L, et al. Updated methodological guidance for the conduct of scoping reviews. JBI Evid Synth. 2020;18(10):2119–26.

12. Tricco AC, Lillie E, Zarin W, O'Brien KK, Colquhoun H, Levac D, et al. PRISMA Extension for Scoping Reviews (PRISMA-ScR): Checklist and Explanation. Ann Intern Med. 2018 Oct;169(7):467–73.

13. Arksey H, O'Malley L. Scoping studies: Towards a methodological framework. Int J Soc Res Methodol Theory Pract. 2005;8(1):19–32.

14. Levac D, Colquhoun H, O'Brien KK. Scoping studies: advancing the methodology. Implement Sci. 2010;5:69.

For peer review only

15. Li J. Digital technologies for mental health improvements in the COVID-19 pandemic: a scoping review. BMC Public Health. 2023;23(1):1–10.

16. Veritas Health Innovation. Covidence systematic review software [Internet]. Melbourne, Australia; Available from: www.covidence.org

For peer review only

Supplemental file 1 - Preferred Reporting Items for Systematic reviews and Meta-Analyses extension for Scoping Reviews (PRISMA-ScR) Checklist

SECTION	ITEM	PRISMA-ScR CHECKLIST ITEM	REPORTED ON PAGE #
**TITLE**			
Title	1	Identify the report as a scoping review.	1
**ABSTRACT**			
Structured summary	2	Provide a structured summary that includes (as applicable): background, objectives, eligibility criteria, sources of evidence, charting methods, results, and conclusions that relate to the review questions and objectives.	1
**INTRODUCTION**			
Rationale	3	Describe the rationale for the review in the context of what is already known. Explain why the review questions/objectives lend themselves to a scoping review approach.	3
Objectives	4	Provide an explicit statement of the questions and objectives being addressed with reference to their key elements (e.g., population or participants, concepts, and context) or other relevant key elements used to conceptualize the review questions and/or objectives.	5
**METHODS**			
Protocol and registration	5	Indicate whether a review protocol exists; state if and where it can be accessed (e.g., a Web address); and if available, provide registration information, including the registration number.	2
Eligibility criteria	6	Specify characteristics of the sources of evidence used as eligibility criteria (e.g., years considered, language, and publication status), and provide a rationale.	7
Information sources*	7	Describe all information sources in the search (e.g., databases with dates of coverage and contact with authors to identify additional sources), as well as the date the most recent search was executed.	5
Search	8	Present the full electronic search strategy for at least 1 database, including any limits used, such that it could be repeated.	Supplementary file 1
Selection of sources of evidence†	9	State the process for selecting sources of evidence (i.e., screening and eligibility) included in the scoping review.	7
Data charting process‡	10	Describe the methods of charting data from the included sources of evidence (e.g., calibrated forms or forms that have been tested by the team before their use, and whether data charting was done independently or in duplicate) and any processes for obtaining and confirming data from investigators.	7
Data items	11	List and define all variables for which data were sought and any assumptions and simplifications made.	N/A
Critical appraisal of individual sources of evidence§	12	If done, provide a rationale for conducting a critical appraisal of included sources of evidence; describe the methods used and how this information was used in any data synthesis (if appropriate).	N/A

For peer review only - http://bmjopen.bmj.com/site/about/guidelines.xhtml

SECTION	ITEM	PRISMA-ScR CHECKLIST ITEM	REPORTED ON PAGE #
Synthesis of results	13	Describe the methods of handling and summarizing the data that were charted.	N/A
**RESULTS**			
Selection of sources of evidence	14	Give numbers of sources of evidence screened, assessed for eligibility, and included in the review, with reasons for exclusions at each stage, ideally using a flow diagram.	N/A
Characteristics of sources of evidence	15	For each source of evidence, present characteristics for which data were charted and provide the citations.	N/A
Critical appraisal within sources of evidence	16	If done, present data on critical appraisal of included sources of evidence (see item 12).	N/A
Results of individual sources of evidence	17	For each included source of evidence, present the relevant data that were charted that relate to the review questions and objectives.	N/A
Synthesis of results	18	Summarize and/or present the charting results as they relate to the review questions and objectives.	N/A
**DISCUSSION**			
Summary of evidence	19	Summarize the main results (including an overview of concepts, themes, and types of evidence available), link to the review questions and objectives, and consider the relevance to key groups.	N/A
Limitations	20	Discuss the limitations of the scoping review process.	N/A
Conclusions	21	Provide a general interpretation of the results with respect to the review questions and objectives, as well as potential implications and/or next steps.	N/A
**FUNDING**			
Funding	22	Describe sources of funding for the included sources of evidence, as well as sources of funding for the scoping review. Describe the role of the funders of the scoping review.	8

JBI = Joanna Briggs Institute; PRISMA-ScR = Preferred Reporting Items for Systematic reviews and Meta-Analyses extension for Scoping Reviews.
* Where *sources of evidence* (see second footnote) are compiled from, such as bibliographic databases, social media platforms, and Web sites.
† A more inclusive/heterogeneous term used to account for the different types of evidence or data sources (e.g., quantitative and/or qualitative research, expert opinion, and policy documents) that may be eligible in a scoping review as opposed to only studies. This is not to be confused with *information sources* (see first footnote).
‡ The frameworks by Arksey and O'Malley (6) and Levac and colleagues (7) and the JBI guidance (4, 5) refer to the process of data extraction in a scoping review as data charting.
§ The process of systematically examining research evidence to assess its validity, results, and relevance before using it to inform a decision. This term is used for items 12 and 19 instead of "risk of bias" (which is more applicable to systematic reviews of interventions) to include and acknowledge the various sources of evidence that may be used in a scoping review (e.g., quantitative and/or qualitative research, expert opinion, and policy document).

From: Tricco AC, Lillie E, Zarin W, O'Brien KK, Colquhoun H, Levac D, et al. PRISMA Extension for Scoping Reviews (PRISMAScR): Checklist and Explanation. Ann Intern Med. 2018;169:467–473. doi: 10.7326/M18-0850.

For peer review only - http://bmjopen.bmj.com/site/about/guidelines.xhtml

Supplemental file 2

Search of academic database

Pubmed Search

ALL FIELDS Search: ((((("well-being" OR wellbeing OR stress OR "mental disorder" OR "mental illness" OR "mental health" OR depress* OR "psychological health" OR anxiety OR psychiatric OR "mood disorder" OR "mental disease") AND (youth* OR young* OR child* OR adolescen* OR student* OR teen*)) AND (marginali* OR disadvan* OR vulnerab* OR depriv* OR "ethnic minorit*" OR immigra* OR homeless OR minorit* OR "low-income" OR disabili* OR isolat* OR LGBTQ+ OR NEET OR NEETs)) AND (intervention OR promo* OR prevent* OR program* OR support OR polic* OR implementation OR evaluation OR therap* OR develop*)) AND (digital* OR mHealth OR eHealth OR "web-based" OR "internet-based" OR "mobile phone" OR "text message" OR "text-based" OR SMS OR app OR "artificial intelligence" OR tele* OR computeri* OR online OR "electronic health" OR "telemedicine")) AND ("co-produ*" OR "co-design*" OR "youth-led" OR participatory OR collab*) Filters: from 2021 – 2023; **309 returns**

ALL FIELDS Search: ("well-being" OR wellbeing OR stress OR "mental disorder" OR "mental illness" OR "mental health" OR depress* OR "psychological health" OR anxiety OR psychiatric OR "mood disorder" OR "mental disease") AND (youth* OR young* OR child* OR adolescen* OR student* OR teen*) AND (marginali* OR disadvan* OR vulnerab* OR depriv* OR "ethnic minorit*" OR immigra* OR homeless OR minorit* OR "low-income" OR disabili* OR isolat* OR LGBTQ+ OR NEET OR NEETs OR refugee OR indigenous OR neurodiver*) AND (intervention OR promo* OR prevent* OR program* OR support OR polic* OR implementation OR evaluation OR therap* OR develop*) AND (digital* OR mHealth OR eHealth OR "web-based" OR "internet-based" OR "mobile phone" OR "text message" OR "text-based" OR SMS OR app OR "artificial intelligence" OR tele* OR computeri* OR online OR "electronic health" OR "telemedicine") AND ("co-produ*" OR "co-design*" OR "youth-led" OR participatory OR collab*) Filters: from 2021/1/1 - 2023/10/23; 350 returns

For peer review only

Supplemental file 3

Template for charting included articles

**Reference**	
**Country of study**	
**Focus of paper: Co Production in design, implementation, evaluation or overall evaluation of co-designing digital mental health intervention**	
**Population; target issue; brief account of digital intervention; mental health promotion-primary prevention or targeted intervention**	
**Methodology of co-design; sample; outcomes**	
**Key Theme(s)**	
**Issues/ debates identified**	

For peer review only

Limitations	
**Evaluation of study (key findings / overall conclusions only)**	
**Relevance to research objective/s**	
**Notes:**	
**Reviewed by:**	

* This template will be further refined at the review stage and updated accordingly.

For peer review only

BMJ Open

Co-production of accessible digital mental health supports in partnership with young people from marginalised backgrounds: a scoping review protocol

Journal:	*BMJ Open*
Manuscript ID	bmjopen-2023-082247.R2
Article Type:	Protocol
Date Submitted by the Author:	11-Apr-2024
Complete List of Authors:	Kealy, Carmen; University of Galway<br>Potts, Courtney; Ulster University<br>Mulvenna, Maurice; University of Ulster, School of Computing<br>Donohoe, Gary; University of Galway<br>O'Neill, Siobhan; Ulster University, Psychology<br>Barry, Margaret M.; University of Galway
**Primary Subject Heading**:	Mental health
Secondary Subject Heading:	Qualitative research, Health services research
Keywords:	Adolescent, MENTAL HEALTH, eHealth, Internet

SCHOLARONE™
Manuscripts

Co-production of accessible digital mental health supports in partnership with young people from marginalised backgrounds: a scoping review protocol

Carmen Kealy[1], Courtney Potts[2]*, Maurice D. Mulvenna[3], Gary Donohoe[4], Siobhán O'Neill[2] and Margaret M. Barry[1]

[1] Health Promotion Research Centre, University of Galway, Ireland

[2] School of Psychology, Ulster University, Northern Ireland

[3] School of Computing, Ulster University, Northern Ireland

[4] School of Psychology, University of Galway, Ireland

*corresponding author: c.potts@ulster.ac.uk, 2-24 York Street, Belfast, BT15 1AP, tel: +442895367509

Word count: 2,203

Keywords

Adolescent, Mental Health, eHealth, Internet

Abstract

Introduction Despite evidence supporting the value of digital supports for enhancing youth mental health services, there is a lack of guidance on how best to engage with young people in co-production processes during the design and evaluation of these technologies. User input is crucial in digital mental health, especially for disadvantaged, vulnerable, and marginalised young people as they are often excluded from co-production. A scoping review of international literature written in English will explore co-production processes with marginalised young people in digital mental health supports, from mental health promotion to targeted interventions. The review is guided by the research question: what are the most appropriate co-production processes for engaging young people, especially marginalised young people, in the different stages of designing and evaluating digital mental health supports? The review aims to map and summarise evidence, inform the overall research project, and address knowledge gaps.

Methods and analysis The scoping review utilises Arksey and O'Malley's framework and the Preferred Reporting Items for Systematic Reviews and Meta-analysis Protocols Extension for Scoping Reviews (PRISMA-ScR). From 22-24 October 2023, PubMed, Scopus, EBSCO, ASSIA, Web of Science, Ovid Medline, Cochrane database, Embase, Google Scholar, ProQuest, OAIster and BASE will be systematically searched. Papers from 2021 onwards with a range of study designs and evidence that illustrate engagement with marginalised young people (aged 16-25) in design, implementation, and evaluation of digital technologies for young people's mental health, will be considered for inclusion. At least two reviewers will

For peer review only - http://bmjopen.bmj.com/site/about/guidelines.xhtml

screen full texts and chart data. The results of this review will be summarised quantitatively through numerical counts of included literature and qualitatively through a narrative synthesis.

Ethics and dissemination Ethics approval is not required. Results will be disseminated through publications in peer reviewed journals.

Registration This scoping review protocol has been registered with the Open Science Framework (https://osf.io/9xhgv).

ARTICLE SUMMARY

Strengths and limitations of this study

- This review will explore the literature on co-production across all stages of design and evaluation of digital mental health supports for young people, with a particular emphasis on marginalised young people who are often excluded from these processes.
- The review will be limited to studies published from 2021 onwards to capture the most current literature on the use of co-production methods and the dynamic nature of this topic.
- Due to the scoping review design, quality assessments of the included articles will not be performed.
- The synthesis of knowledge will serve as a basis for the wider research project and concrete calls to action.

For peer review only - http://bmjopen.bmj.com/site/about/guidelines.xhtml

INTRODUCTION

There is increasing evidence that some digital mental health supports may improve the accessibility of mental health services and supports for young people, including marginalised young people with mental health needs who would otherwise unlikely seek help (1). Guidelines with regard to digital mental health interventions highlight the importance of early user input in the development, implementation, and evaluation of technologies to ensure that they are engaging, feasible, acceptable, and potentially effective (2). However, there is a lack of guidance concerning the most appropriate co-production processes for engaging young people in the different stages of assessing digital mental health technologies, especially with regard to those who are disadvantaged, vulnerable and /or marginalised (3-5).

Focusing on innovative digital mental health approaches led by young people, this review will consider co-production in the design, development, implementation, and evaluation phases of digital mental health supports for marginalised young people, as well as overall evaluations of co-production processes in this context. The review is part of a larger project, called 'Atlantic Futures' (6). One of the work packages (Research Stream 4), within this project explores accessible digital supports and blended services to promote youth mental health on a shared island basis in Ireland and Northern Ireland. This is to be achieved through engaging with young people (aged 16-25), including those who are marginalised, and the staff who support them in health, youth, and community services.

Marginalisation

This scoping review focuses on young people categorised as 'marginalised' in the digital mental health support context. A briefing paper produced by UNICEF's Office of Research defines "disadvantaged, vulnerable and/or marginalised adolescents as individuals aged 10–19, who are excluded from social, economic and/or educational opportunities enjoyed by other adolescents in their community due to numerous factors beyond their control" (7). These factors include those operating at the social level (such as economic inequality, violence, stigma, racism, migration), family level (including neglect and abuse) and individual level (e.g., disability, ethnicity). Examples for disadvantaged, vulnerable and/or marginalised young people include immigrants, refugees, orphans, and those who belong to stigmatised indigenous, ethnic, or religious groups. They also include individuals who identify as belonging to sexual minorities (e.g., gay, lesbian, bisexual, queer) or gender minorities (e.g., transgender, gender diverse).

Previous reviews on co-production in digital mental health interventions have highlighted either an underrepresentation of young people with learning disabilities and specific difficulties (3) or a marked absence of young people including, but not limited to those who are migrants, asylum seekers and refugees, those experiencing homelessness but also those from socioeconomically deprived backgrounds (8).

In the context of digital mental health technologies, the concept of digital marginalisation is also highly relevant, and this typically refers to people who do not have or use the internet, who lack access to fast and reliable internet connection (e.g., people living in rural areas), and who lack the skills or the access to developing skills for using the internet (8). Many marginalised groups face barriers in accessing online services, which can further perpetuate

For peer review only - http://bmjopen.bmj.com/site/about/guidelines.xhtml

the "digital divide," a technology-based form of social inequality that can increase marginalisation in other areas of their lives (9).

Considering the above, the project seeks to take into account the specific needs of young people who are disadvantaged, vulnerable and /or marginalised, from a socio-economic, cultural, and digital perspective.

Thus, in order to investigate co-production and marginalisation, the scoping review will include, but not be limited to, the following groups of young people, who have been identified as marginalised in the Irish context

- Living in isolated rural areas (10)
- Socioeconomically deprived
- Unemployed
- Not in education, employment, or training (NEET)
- Immigrants
- Asylum seekers
- Refugees
- Travelling community
- LGBTQ+ community
- Living with a disability
- Homeless

This scoping review intends to explore the most appropriate co-production processes for engaging young people in the different stages of designing, developing, implementing, and evaluating digital mental health technologies, especially with regard to those who are marginalised. Due to the exploratory nature of this research question, a scoping review approach has been selected to explore the breadth and depth of the extant literature on co-production processes with marginalised young people in the context of digital mental health supports, ranging from mental health promotion, and primary prevention to targeted interventions. This includes all types of digital interventions, such as websites and apps but not studies that report on digital delivery of services that are typically administered face-to-face such as remote counselling. The review aims to map and summarise the evidence, inform the overall research project, as well as identify and address knowledge gaps (11).

METHODS AND ANALYSIS

This scoping review is prepared in accordance with the Preferred Reporting Items for Systematic Reviews and Meta-analysis Protocols Extension for Scoping Reviews guidelines (PRISMA-ScR) (12) (Supplemental file 1). A scoping review is a systematic way of exploring a topic area, in which main concepts and knowledge gaps are identified within a developing field of research (13). In contrast to systematic reviews, the research questions for a scoping review are frequently broader and more exploratory (13), and hence appropriate for this topic due to insufficient research and a lack of synthesised knowledge. Guided by Arksey and O'Malley's (13) five-step set, but also Levac and colleagues (14) update of this framework, the review process will follow this tentative timeline:

1. Identifying the research question—completed in October 2023

For peer review only - http://bmjopen.bmj.com/site/about/guidelines.xhtml

2. Identifying relevant studies—completed in October 2023

3. Study selection—November-December 2023

4. Charting the data—January 2024.

5. Collating, summarising, and reporting the results—February 2024

Identifying the research question

In keeping with the exploratory nature of scoping reviews, the research team identified one main research question: what are the most appropriate co-production processes for engaging young people in the different stages of designing and assessing digital mental health technologies, especially regarding those who are marginalised?

Identifying relevant studies

Following a preliminary search to see the volume returned, the following electronic databases will be systematically searched for international literature published in English: PubMed, Scopus, EBSCO (CINAHL/PsycINFO/PsycArticles), ASSIA (Applied Social Sciences Index & Abstracts), Web of Science, Ovid Medline, Cochrane database, Embase, Google Scholar, ProQuest Dissertations & Theses A&I, OAIster (Catalog of Open Access Resources) and BASE (Bielefeld Academic Search Engine). The focus of this scoping review is informed by previous reviews on co-production and/or marginalised young people in digital mental health interventions (1-5), which have been conducted and/or published since the onset of the global Coronavirus pandemic and associated restrictions in March 2020. Highlighting an underrepresentation of marginalised young people, searches will lead on from these reviews and be limited from January 2021 to the date of search commencement in October 2023, to ensure that the review reflects the most current literature on the use of co-production methods and thus the nature of this dynamic research topic. Published work from 2021 onwards is included to mirror the current state of literature more accurately, given the accelerated use and assessment of digital mental health technologies throughout the sector during the pandemic (15).

Title, abstract and keyword fields will be searched with the following search terms (Table 1) where advanced search options are possible.

Table 1: Search terms

Mental health	“well-being” OR wellbeing OR stress OR “mental disorder” OR “mental illness” OR “mental health” OR depress* OR “psychological health” OR anxiety OR psychiatric OR “mood disorder” OR “mental disease”
Young people	youth* OR young* OR child* OR adolescen* OR student* OR teen*

For peer review only - http://bmjopen.bmj.com/site/about/guidelines.xhtml

Marginalisation*	marginali* OR disadvan* OR vulnerab* OR depriv* OR "ethnic minorit*" OR immigra* OR homeless OR minorit* OR "low-income" OR disabili* OR isolat* OR LGBTQ+ OR NEET OR NEETs OR refugee OR indigenous OR neurodiver*
Interventions	Intervention OR promo* OR prevent* OR program* OR support OR polic* OR implementation OR evaluation OR therap* OR develop*
Technology	digital* OR mHealth OR eHealth OR "web-based" OR "internet-based" OR "mobile phone" OR "text message" OR "text-based" OR SMS OR app OR "artificial intelligence" OR tele* OR computeri* OR online OR "electronic health" OR "telemedicine"
Co-production	"co-produ*" OR "co-design*" OR "youth-led" OR participatory OR collab*

*Search terms for the category marginalisation consist of definition terms provided by UNICEF's briefing paper (9) as well as search terms found adequate to capture the groups of young people identified as marginalized.in the Irish but also international context.

For Google Scholar and BASE (Bielefeld Academic Search Engine) the following search terms will be used: (Co-production OR Co-design) AND (Digital mental health) AND (marginal*youth* OR disadvant* adolescen* OR vulnerable teen* OR support* OR intervention OR promot* OR prevent* OR develop* OR evalua* OR implem*).

An example of the electronic search strategy for PubMed has been included (supplemental file 2)

Results from academic and grey literature databases will be downloaded in RIS format and uploaded to the Covidence software (16), a screening and data extraction tool for conducting reviews, which allows screening to be more efficient and easily tracked.

Study selection

Two senior research team members will be assigned the role of reviewers and will screen all articles identified from the databases independently and 20% of the overall article returns will also be screened by two other members of the research team. Titles and abstracts will be reviewed based on the initial search and will be included in accordance with the eligibility criteria described below. Duplicate articles will be removed, while full texts will be examined to create a final list of included studies. Any disagreements on the inclusion of any articles will be resolved through discussion and in consultation with other team members if necessary.

For peer review only - http://bmjopen.bmj.com/site/about/guidelines.xhtml

The reference lists of the included papers will be examined as well to verify that all relevant publications are incorporated. As the review develops, other techniques for searching may be implemented, and any additions or changes will be noted.

Eligibility Criteria

Empirical research and discussions in the literature will be included in the review if they: (1) are published since January 2021; (2) are published in English; (3) focus on co-production/co-design of digital youth mental health supports with marginalised young people aged 16-25; and (4) utilise innovative youth-led methodologies to design, develop, implement and/or evaluate digital mental health supports for young people.

Articles will NOT be included if they are: (1) non-digital interventions; (2) technologies developed primarily for physical health or diagnostic, screening, monitoring, communication or data management tools; (3) focus on digital mental health supports without young people's input in the development, implementation and/ or evaluation of these supports; and (4) involving children (under the age of 16) and adults (over the age of 25).

Charting the data

Data from the identified studies will be collected and charted according to key groupings, e.g., based on the focus of the article – design, development, implementation, evaluation. From each eligible article, the reviewers will include authorship, year and journal of publication, the general characteristics of participants (i.e., age, gender, and sociodemographic information), geographic location of study, study methods, the identified co-production processes for digital mental health supports, limitations, and any other key findings relating to the research question. An example of a charting template has been included (supplemental file 3). A combination of Excel and Covidence management software will be used to collate the charted data and to manage the screening process.

Collating, summarising and reporting the results

As this scoping review is intended to provide an overview of the existing literature, and not to critically appraise the included articles, risk of bias will not be assessed. However, we will include a discussion of any limitations found in the included body of evidence. The results of this scoping review will be summarised quantitatively by highlighting the amount and type of studies reviewed, and qualitatively through a narrative synthesis. Tables and/or charts will be used to map the study findings and provide an overview, and the screening process will be visualised through a flowchart. Given the exploratory nature of this study, any factors related to co-producing digital mental health supports will be considered for review. Of particular interest is the sourcing of state-of-the-art examples that engage with marginalised young people (aged 16-25 and capturing the out-of-school population of young people in Ireland as per education policies) in an innovative way, not only in the initial exploration/design but also implementation and evaluation phase of digital technologies for young people's mental health. The review also seeks to identify published works that have evaluated overall co-production processes in the creation of digital mental health supports for young people.

Patient and public involvement

For peer review only

For peer review only - http://bmjopen.bmj.com/site/about/guidelines.xhtml

The design of this scoping review protocol did not involve patients or the public.

Ethics and dissemination

As this scoping review is intended to synthesise the current breadth of knowledge on co-production processes with marginalised youth in the context of digital mental health supports, no ethics approval is required. The results of this scoping review will be disseminated through publications in peer-reviewed journals.

DISCUSSION

The results of this study will establish what is currently known about the most appropriate co-production processes for engaging young people in the different stages of assessing digital technologies, especially with regard to those who are disadvantaged, vulnerable and /or marginalised. To the best of our knowledge, this scoping review protocol is the first on this topic and will identify key themes and gaps in understanding how to engage with marginalised young people (aged 16-25) in an innovative and meaningful way when designing/ developing, as well as implementing and evaluating, digital mental health technologies for young people's mental health. The results will serve as the basis for the wider project, including: a qualitative exploration of digital mental health challenges facing young people across the island of Ireland; identifying, with young people, appropriate digital mental health apps and interventions; validating, in partnership with young people, what digital mental health interventions work; and using these findings to inform policy and practice recommendations for health providers across the island of Ireland and beyond. The review will contribute to existing literature in the field by not only highlighting what has been achieved to date but also by proposing potential ways of advancing the inclusion of marginalised young people in the co-production of digital mental health supports. This will ensure user input, access, and subsequent improvement in digital mental health technologies.

AUTHOR STATEMENT

Author contributors CK, CP, MDM, GD, SON and MMB contributed to the development of this manuscript. CK conceptualised the research question, designed the study and prepared the first draft of the manuscript. MMB helped refine the research question and provide review expertise. All authors contributed to the refining of the study design, as well as to the editing and revising of this protocol. All authors have approved the final manuscript for submission.

Funding Atlantic Futures is a €4 million four year cross-border research project funded by The North-South Research Programme (422560). The North-South Research Programme is a collaborative scheme funded through the Government's Shared Island Fund. It is administered by the Higher Education Authority (HEA) on behalf of the Department of Further and Higher Education, Research, Innovation and Science.

Competing interests None declared.

Patient consent for publication Not required.

For peer review only - http://bmjopen.bmj.com/site/about/guidelines.xhtml

REFERENCES

1. Piers R, Williams JM, Sharpe H. Review: Can digital mental health interventions bridge the 'digital divide' for socioeconomically and digitally marginalised youth? A systematic review. Child Adolesc Ment Health. 2023;28(1):90–104.

2. Liverpool S, Mota CP, Sales CMD, Čuš A, Carletto S, Hancheva C, et al. Engaging children and young people in digital mental health interventions: Systematic review of modes of delivery, facilitators, and barriers. J Med Internet Res. 2020;22(6):e16317. doi: 10.2196/16317

3. Bevan Jones R, Stallard P, Agha SS, Rice S, Werner-Seidler A, Stasiak K, et al. Practitioner review: Co-design of digital mental health technologies with children and young people. J Child Psychol Psychiatry Allied Discip. 2020;61(8):928–40.

4. Bear HA, Nunes LA, DeJesus J, Liverpool S, Moltrecht B, Neelakantan L, et al. Determination of Markers of Successful Implementation of Mental Health Apps for Young People: Systematic Review. J Med Internet Res. 2022;24(11):1–17.

5. Maxwell N, Corliss C. Implementing co-productive practice with public services: Messages from the research [online] 2020. Available from: https://www.violencepreventionwales.co.uk/cms-assets/research/Implementing-co-productive-practice-with-public-services.pdf (accessed 8 February 2024).

6. Atlantic Futures Project [online] https://www.atlanticfutures.com/ (accessed 8 February 2024).

7. Auerswald CL, Piatt AA, Mirzazadeh A. Research with Disadvantaged, Vulnerable and/or Marginalized Adolescents [online] Innocenti Research Briefs. Innocenti, Florence; 2017. https://www.unicef-irc.org/publications/878-research-with-disadvantaged-vulnerable-and-or-marginalized-adolescents.html (accessed 8 February 2024).

8. Eickers G, Rath M. Digital Change and Marginalized Communities: Changing Attitudes Towards Digital Media in the Margins. ICERI2021 Proc. 2021;1:5286–93.

9. Sin J, Franz RL, Munteanu C, Neves BB. Digital design marginalization: New perspectives on designing inclusive interfaces. In: Conference on Human Factors in Computing Systems - Proceedings. Yokohama, Japan: ACM PUB27 New York, NY, USA; 2021.

10. Pobal Maps [online] https://maps.pobal.ie/WebApps/DeprivationIndices/index.html (accessed 8 February 2024).

11. Peters MDJ, Marnie C, Tricco AC, Pollock D, Munn Z, Alexander L, et al. Updated methodological guidance for the conduct of scoping reviews. JBI Evid Synth. 2020;18(10):2119–26.

12. Tricco AC, Lillie E, Zarin W, O'Brien KK, Colquhoun H, Levac D, et al. PRISMA Extension for Scoping Reviews (PRISMA-ScR): Checklist and Explanation. Ann Intern Med. 2018 Oct;169(7):467–73.

13. Arksey H, O'Malley L. Scoping studies: Towards a methodological framework. Int J Soc Res Methodol Theory Pract. 2005;8(1):19–32.

For peer review only - http://bmjopen.bmj.com/site/about/guidelines.xhtml

14. Levac D, Colquhoun H, O'Brien KK. Scoping studies: advancing the methodology. Implement Sci. 2010;5:69.

15. Li J. Digital technologies for mental health improvements in the COVID-19 pandemic: a scoping review. BMC Public Health. 2023;23(1):1–10.

16. Veritas Health Innovation. Covidence systematic review software [online]. Melbourne, Australia. www.covidence.org (accessed 8 February 2024).

For peer review only

For peer review only - http://bmjopen.bmj.com/site/about/guidelines.xhtml

Supplemental file 1 - Preferred Reporting Items for Systematic reviews and Meta-Analyses extension for Scoping Reviews (PRISMA-ScR) Checklist

SECTION	ITEM	PRISMA-ScR CHECKLIST ITEM	REPORTED ON PAGE #
**TITLE**			
Title	1	Identify the report as a scoping review.	1
**ABSTRACT**			
Structured summary	2	Provide a structured summary that includes (as applicable): background, objectives, eligibility criteria, sources of evidence, charting methods, results, and conclusions that relate to the review questions and objectives.	1
**INTRODUCTION**			
Rationale	3	Describe the rationale for the review in the context of what is already known. Explain why the review questions/objectives lend themselves to a scoping review approach.	3
Objectives	4	Provide an explicit statement of the questions and objectives being addressed with reference to their key elements (e.g., population or participants, concepts, and context) or other relevant key elements used to conceptualize the review questions and/or objectives.	5
**METHODS**			
Protocol and registration	5	Indicate whether a review protocol exists; state if and where it can be accessed (e.g., a Web address); and if available, provide registration information, including the registration number.	2
Eligibility criteria	6	Specify characteristics of the sources of evidence used as eligibility criteria (e.g., years considered, language, and publication status), and provide a rationale.	7
Information sources*	7	Describe all information sources in the search (e.g., databases with dates of coverage and contact with authors to identify additional sources), as well as the date the most recent search was executed.	5
Search	8	Present the full electronic search strategy for at least 1 database, including any limits used, such that it could be repeated.	Supplementary file 1
Selection of sources of evidence†	9	State the process for selecting sources of evidence (i.e., screening and eligibility) included in the scoping review.	7
Data charting process‡	10	Describe the methods of charting data from the included sources of evidence (e.g., calibrated forms or forms that have been tested by the team before their use, and whether data charting was done independently or in duplicate) and any processes for obtaining and confirming data from investigators.	7
Data items	11	List and define all variables for which data were sought and any assumptions and simplifications made.	N/A
Critical appraisal of individual sources of evidence§	12	If done, provide a rationale for conducting a critical appraisal of included sources of evidence; describe the methods used and how this information was used in any data synthesis (if appropriate).	N/A

St. Michael's
Inspired Care.
Inspiring Science.

SECTION	ITE M	PRISMA-ScR CHECKLIST ITEM	REPORTED ON PAGE #
Synthesis of results	13	Describe the methods of handling and summarizing the data that were charted.	N/A
**RESULTS**			
Selection of sources of evidence	14	Give numbers of sources of evidence screened, assessed for eligibility, and included in the review, with reasons for exclusions at each stage, ideally using a flow diagram.	N/A
Characteristics of sources of evidence	15	For each source of evidence, present characteristics for which data were charted and provide the citations.	N/A
Critical appraisal within sources of evidence	16	If done, present data on critical appraisal of included sources of evidence (see item 12).	N/A
Results of individual sources of evidence	17	For each included source of evidence, present the relevant data that were charted that relate to the review questions and objectives.	N/A
Synthesis of results	18	Summarize and/or present the charting results as they relate to the review questions and objectives.	N/A
**DISCUSSION**			
Summary of evidence	19	Summarize the main results (including an overview of concepts, themes, and types of evidence available), link to the review questions and objectives, and consider the relevance to key groups.	N/A
Limitations	20	Discuss the limitations of the scoping review process.	N/A
Conclusions	21	Provide a general interpretation of the results with respect to the review questions and objectives, as well as potential implications and/or next steps.	N/A
**FUNDING**			
Funding	22	Describe sources of funding for the included sources of evidence, as well as sources of funding for the scoping review. Describe the role of the funders of the scoping review.	8

JBI = Joanna Briggs Institute; PRISMA-ScR = Preferred Reporting Items for Systematic reviews and Meta-Analyses extension for Scoping Reviews.
* Where *sources of evidence* (see second footnote) are compiled from, such as bibliographic databases, social media platforms, and Web sites.
† A more inclusive/heterogeneous term used to account for the different types of evidence or data sources (e.g., quantitative and/or qualitative research, expert opinion, and policy documents) that may be eligible in a scoping review as opposed to only studies. This is not to be confused with *information sources* (see first footnote).
‡ The frameworks by Arksey and O'Malley (6) and Levac and colleagues (7) and the JBI guidance (4, 5) refer to the process of data extraction in a scoping review as data charting.
§ The process of systematically examining research evidence to assess its validity, results, and relevance before using it to inform a decision. This term is used for items 12 and 19 instead of "risk of bias" (which is more applicable to systematic reviews of interventions) to include and acknowledge the various sources of evidence that may be used in a scoping review (e.g., quantitative and/or qualitative research, expert opinion, and policy document).

From: Tricco AC, Lillie E, Zarin W, O'Brien KK, Colquhoun H, Levac D, et al. PRISMA Extension for Scoping Reviews (PRISMAScR): Checklist and Explanation. Ann Intern Med. 2018;169:467–473. doi: 10.7326/M18-0850.

Supplemental file 2

Search of academic database

Pubmed Search

ALL FIELDS Search: ((((("well-being" OR wellbeing OR stress OR "mental disorder" OR "mental illness" OR "mental health" OR depress* OR "psychological health" OR anxiety OR psychiatric OR "mood disorder" OR "mental disease") AND (youth* OR young* OR child* OR adolescen* OR student* OR teen*)) AND (marginali* OR disadvan* OR vulnerab* OR depriv* OR "ethnic minorit*" OR immigra* OR homeless OR minorit* OR "low-income" OR disabili* OR isolat* OR LGBTQ+ OR NEET OR NEETs)) AND (intervention OR promo* OR prevent* OR program* OR support OR polic* OR implementation OR evaluation OR therap* OR develop*)) AND (digital* OR mHealth OR eHealth OR "web-based" OR "internet-based" OR "mobile phone" OR "text message" OR "text-based" OR SMS OR app OR "artificial intelligence" OR tele* OR computeri* OR online OR "electronic health" OR "telemedicine")) AND ("co-produ*" OR "co-design*" OR "youth-led" OR participatory OR collab*) Filters: from 2021 – 2023; **309 returns**

ALL FIELDS Search: ("well-being" OR wellbeing OR stress OR "mental disorder" OR "mental illness" OR "mental health" OR depress* OR "psychological health" OR anxiety OR psychiatric OR "mood disorder" OR "mental disease") AND (youth* OR young* OR child* OR adolescen* OR student* OR teen*) AND (marginali* OR disadvan* OR vulnerab* OR depriv* OR "ethnic minorit*" OR immigra* OR homeless OR minorit* OR "low-income" OR disabili* OR isolat* OR LGBTQ+ OR NEET OR NEETs OR refugee OR indigenous OR neurodiver*) AND (intervention OR promo* OR prevent* OR program* OR support OR polic* OR implementation OR evaluation OR therap* OR develop*) AND (digital* OR mHealth OR eHealth OR "web-based" OR "internet-based" OR "mobile phone" OR "text message" OR "text-based" OR SMS OR app OR "artificial intelligence" OR tele* OR computeri* OR online OR "electronic health" OR "telemedicine") AND ("co-produ*" OR "co-design*" OR "youth-led" OR participatory OR collab*) Filters: from 2021/1/1 - 2023/10/23; 350 returns

For peer review only

Supplemental file 3

Template for charting included articles

**Reference**	
**Country of study**	
**Focus of paper: Co Production in design, implementation, evaluation or overall evaluation of co-designing digital mental health intervention**	
**Population; target issue; brief account of digital intervention; mental health promotion-primary prevention or targeted intervention**	
**Methodology of co-design; sample; outcomes**	
**Key Theme(s)**	
**Issues/ debates identified**	

For peer review only

Limitations	
**Evaluation of study (key findings / overall conclusions only)**	
**Relevance to research objective/s**	
**Notes:**	
**Reviewed by:**	

* This template will be further refined at the review stage and updated accordingly.

For peer review only
